# Clinical outcomes and risk factors of non-curative endoscopic submucosal dissection for early gastric cancer: a retrospective multicenter study in Zhejiang, China

**DOI:** 10.3389/fonc.2023.1225702

**Published:** 2023-10-03

**Authors:** Chao-qiong Jin, Jing Zhao, Xiao-yun Ding, Liang-liang Yu, Guo-liang Ye, Xin-jian Zhu, Jian-wei Shen, Ye Yang, Bo Jin, Chun-li Zhang, Bin Lv

**Affiliations:** ^1^ Department of Gastroenterology, First Affiliated Hospital of Zhejiang Chinese Medical University, Hangzhou, China; ^2^ Department of Gastroenterology, Shaoxing People’s Hospital, Shaoxing, Zhejiang, China; ^3^ Department of Gastroenterology, Laboratory of Digestive Diseases, Ningbo First Hospital, Ningbo, China; ^4^ Department of Gastroenterology, Sir Run Run Shaw Hospital Affiliated Hospital of Zhejiang University, Hangzhou, China; ^5^ Department of Gastroenterology, The Affiliated Hospital of Medical School of Ningbo University, Ningbo, China; ^6^ Department of Gastroenterology, Shaoxing Shangyu People’s Hospital, Shaoxing, Zhejiang, China; ^7^ Department of Gastroenterology, Ningbo Medical Center Lihuili Eastern Hospital, Ningbo, China; ^8^ Department of Gastroenterology, HwaMei Hospital, University Of Chinese Academy Of Sciences, Ningbo, China; ^9^ Gastrointestinal Endoscopy Center, First Affiliated Hospital of Zhejiang Chinese Medical University, Hangzhou, China; ^10^ Department of Pathology, First Affiliated Hospital of Zhejiang Chinese Medical University, Hangzhou, China; ^11^ Key Laboratory of Digestive Pathophysiology of Zhejiang Province, First Affiliated Hospital of Zhejiang Chinese Medical University, Hangzhou, China

**Keywords:** early gastric cancer, endoscopic submucosal dissection, lymph node metastasis, cancer recurrence, eCura system, risk factors

## Abstract

**Background:**

Endoscopic submucosal dissection (ESD) for early gastric cancer (EGC) does not always lead to curative resection. Risk factors of lymph node metastasis (LNM)/local cancer residue after non-curative ESD for EGC have not been fully elucidated. We therefore aimed to clarify them and evaluate whether the “eCura system” is reliable for the risk stratification of LNM after non-curative ESD.

**Methods:**

We conducted a multicenter retrospective study at seven institutions in Zhejiang, China, on 128 patients who underwent non-curative ESD for EGC. We divided the patients into two groups according to their therapeutic regimen after non-curative ESD. We analyzed the risk factors for LNM, local cancer residue, cancer recurrence, and cancer-specific mortality. Furthermore, we compared the outcomes in each risk category after applying the “eCura system”.

**Results:**

Among 68 patients undergoing additional surgery, LNM was found in three (4.41%) patients, while local cancer residue was found in eight (11.76%) patients. Multivariate analysis showed that upper third location and deep submucosal invasion were independent risk factors of LNM and local cancer residue. Among 60 patients who underwent simple follow-up, local cancer recurrence was found in four (6.67%) patients and cancer-specific mortality was found in one (1.67%) patient. There were no independent risk factors of cancer recurrence and cancer-specific mortality in our study. During the follow-up period, 5-year overall survival (OS) and disease-free survival (DFS) were 93.8% and 88.9%, respectively. Additionally, LNM and cancer recurrence were significantly associated with the eCura scoring system (p = 0.044 and p = 0.017, respectively), while local cancer residue and cancer-specific mortality were not (p = 0.478 and p = 0.131, respectively).

**Conclusion:**

Clinicians should be aware of the risk factors for the prognosis of patients with non-curative ESD to determine subsequent treatment. Through the application of the “eCura system”, additional surgery should be performed in patients with intermediate/high risk of LNM.

## 1 Introduction

Gastric cancer continues to be a global health problem and is the fifth leading cancer and the third most common cause of cancer-related deaths worldwide (1). The definition of early gastric cancer (EGC) is gastric cancer that invades no deeper than the submucosa, irrespective of lymph node metastasis (LNM) (2). In recent years, endoscopic submucosal dissection (ESD) has become the primary endoscopic treatment for EGC with a low risk of LNM and has shown many advantages in terms of quality of life and short-term and long-term clinical outcomes. Compared with surgery, ESD offers minimally invasive treatment at a lower cost, but with comparable efficacy. However, on the one hand, the precise endoscopic prediction of EGC in terms of tumor depth, lateral spread, or lymphovascular invasion (LVI) is not easy before treatment; on the other hand, the individual skills of physicians and imaging techniques are also a factor. Therefore, cases of non-curative resection still exist.

According to the Japanese Gastric Cancer Treatment Guidelines, non-curative ESD patients with a possible risk of LNM are generally referred for additional gastrectomy with LN dissection (3). However, as LNM occurs in only 5%–10% (4–6) of patients who undergo radical surgery and some patients are unable or unwilling to undergo surgery due to advanced age and basic diseases, it is vital to evaluate the prognosis of a resected lesion that does not meet the curative criteria.

The eCura scoring system (7) was established by Hatta et al. in 2017. They investigated long-term outcomes and validated the risk factors to predict LNM and cancer recurrence after non-curative ESD. The system scored several important clinicopathological factors, including lymphatic invasion, tumor size, positive vertical margin (VM), venous invasion, and submucosal invasion. Patients are categorized into three LNM risk groups according to this scoring system: high (5–7 points: 22.7% risk), intermediate (2–4 points: 6.7% risk), and low (0–1 point: 2.5% risk). The benefits of salvage surgery can be expected in the high-risk group, and follow-up alone might be acceptable in the low-risk group, particularly in elderly patients.

In our study, we investigated the risk factors of LNM, cancer recurrence, cancer-specific mortality, and local cancer residue in patients with non-curative ESD, applied the eCura scoring system to our data, and explored the effectiveness and application value for risk stratification after non-curative ESD for EGC in China.

## 2 Patients and methods

### 2.1 Patients

**Figure 1:**
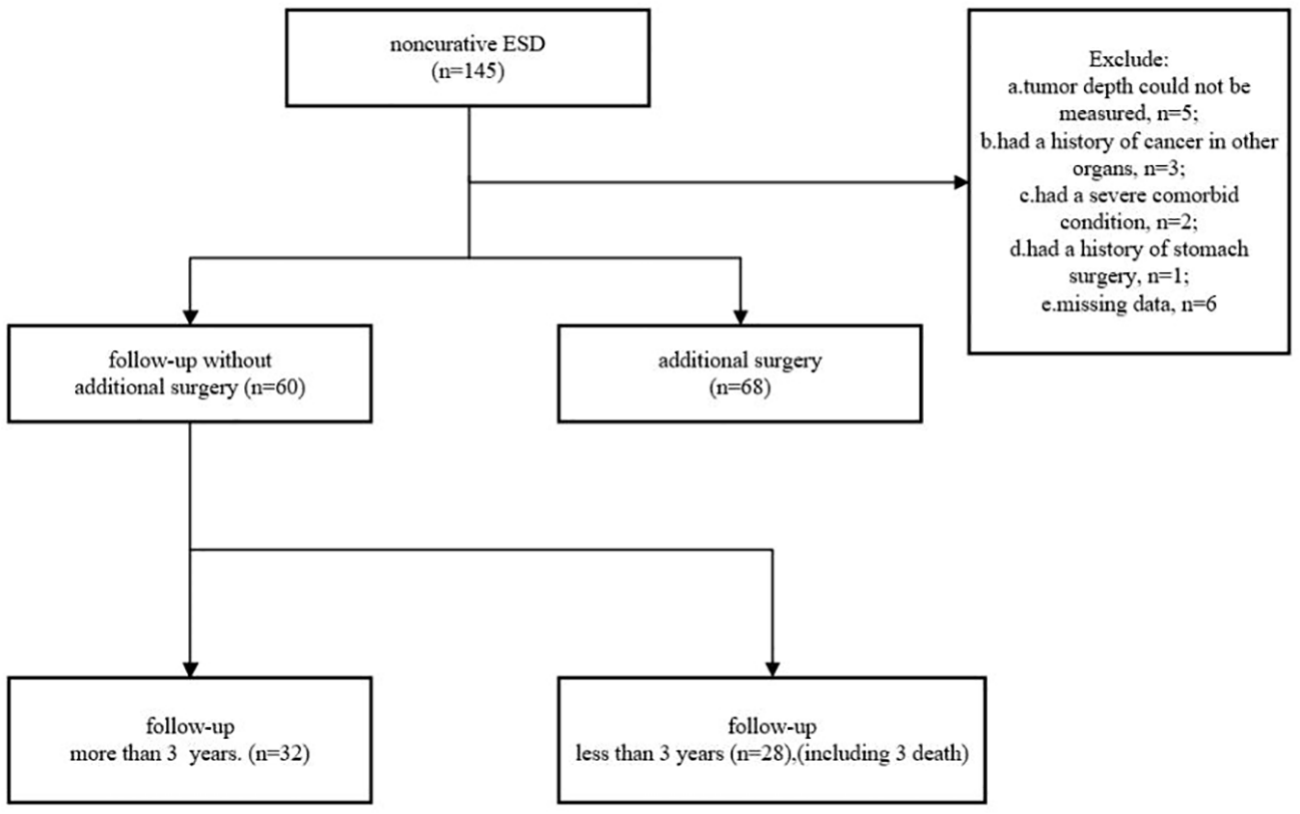
Flowchart of patient enrollment.

We collected data on 145 patients who underwent non-curative ESD for EGC between September 2011 and January 2019 in seven hospitals in Zhejiang Province. Patients were ineligible if they 1) had a history of cancer in other organs, 2) had a history of stomach surgery, 3) had a severe comorbid condition, 4) had a bleeding tendency, 5) were pregnant or possibly pregnant, 6) were unable to provide informed consent, and 7) had missing data. As a result, 17 patients were excluded. A total of 128 patients (97 men and 31 women), comprising 68 undergoing radical surgery and 60 with no additional treatment, were enrolled in this study (**Figure 1**).

### 2.2 Follow-up

Patients who underwent ESD had a scheduled esophagogastroduodenoscopy (EGD) at 3, 6, and 12 months after ESD, and then EGD and CT were recommended once a year. In the group of patients after surgical intervention, EGD and CT were performed at 6 months, and then EGD and abdominal CT were performed once a year. For patients who had not visited our hospital regularly, data were requested from the referring physicians, who contacted the patients at their homes, and statistical data maintained by the local government registry were also examined.

### 2.3 Definitions

Non-curative resection was defined as histological positivity of the resected margins, LVI, or beyond the expanded criteria for ESD (8). Cancer recurrence was defined as tumor relapse in the original and/or other organs after ESD for EGC. Local recurrence was defined as a tumor at a previous ESD site. Cancer-specific mortality included deaths that were identified as being caused by a specific type of cancer. Local cancer residue was defined as a tumor that remained in the original site after surgery.

### 2.4 Assessment of clinicopathological findings

We evaluated each patient for the following factors: age, sex, body mass index, tumor location, tumor size, macroscopic type, depth of tumor invasion, differentiation type, LVI, histological margins, ulceration, and eCura scores.

Tumor location was classified as upper third, middle third, and lower third, and tumors were grouped according to size: up to 20 mm, 20 mm to 30 mm, and more than 30 mm. Well-differentiated or moderately differentiated tubular adenocarcinoma and papillary adenocarcinoma were classified as differentiated carcinoma, whereas poorly differentiated tubular adenocarcinoma, signet-ring cell carcinoma, and mucinous adenocarcinoma were classified as UD carcinoma. The depth of tumor invasion was classified as a mucosal invasion (M), minute SM invasion within 500 μm of the lower margin of the muscularis mucosae (SM1), or submucosal invasion of more than 500 μm from the lower margin of the muscularis mucosae (SM2).

### 2.5 The eCura system

eCura scores were calculated in each patient, as follows: 3 points for lymphatic invasion and 1 point each for tumor size >30 mm, positive VM, venous invasion, and deep submucosal invasion 500 μm (SM2). In this scoring system, patients are categorized into three LNM risk groups based on a score (7).

### 2.6 Statistical analysis

Statistical analysis was performed with Statistical Product and Service Solutions (SPSS) 20.0 statistical software. Fisher’s exact probability test was performed to determine differences in categorical variables between the two groups. Univariate and multivariate analyses were performed with logistic regression analysis. Variables with p < 0.05 in univariate analysis were subjected to multivariate analysis. The 5-year overall survival (OS) and disease-free survival (DFS) were estimated and graphed using the Kaplan–Meier survivor function. In all tests, all p-values were two-sided. A p-value <0.05 was considered statistically significant.

## 3 Results

### 3.1 Characteristics of the patients and lesions

**Figure 1** shows that 32 patients could be followed up for more than 3 years after treatment in the follow-up group. During a median follow-up period of 36 months (range 15–101 months), cancer recurrence was observed in four patients, cancer-specific mortality was found in one (1.67%) patient, and another two died of pneumonia.

**Table 1** shows the clinicopathological findings for the two treatment strategies for 128 non-curative ESD patients. We found that a higher proportion of middle/lower third location (n = 60, 88.24%), tumor size >3 cm (30, 44.12%), SM invasion (56, 82.35%), UD type (21, 30.88%), positive lymphatic invasion (11, 16.18%), and in intermediate/high-risk group (37, 54.41%) were observed in the 68 patients who underwent additional gastrectomy.

**Table 1:** Clinicopathological findings for the two treatment strategies.

	No additional treatment	Radical surgery	p-Value
Age median (years)	66.5 (46, 89)	61.5 (35, 84)	0.037
Sex			
Male	48 (80.00%)	49 (72.06%)	0.295
Female	12 (20.00%)	19 (27.94%)	
Location			
Upper third	17 (28.33%)	8 (11.76%)	0.018
Middle/lower third	43 (71.67)	60 (88.24%)	
Tumor size			
a < 2 cm	27 (45.00%)	19 (27.94%)	0.045
2 cm < a ≤ 3	20 (33.33%)	19 (27.94%)	
a > 3 cm	13 (21.67%)	30 (44.12%)	
Invasion depth			
M	24 (40.00%)	12 (17.65%)	0.005
SM	36 (60.00%)	56 (82.35%)	
Histopathological type			
Differentiated	54 (90.00%)	47 (69.12%)	0.004
undifferentiated	6 (10.00%)	21 (30.88%)	
Lymphatic invasion			
Negative	58 (96.67%)	57 (83.82%)	0.035
Positive	2 (3.33%)	11 (16.18%)	
Vascular invasion			
Negative	54 (90.00%)	56 (82.35%)	0.214
Positive	6 (10.00%)	12 (17.65%)	
Vertical margin			
Negative	48 (80.00%)	46 (67.65%)	0.114
Positive	12 (20.00%)	22 (32.35%)	
H margin			
Negative	36 (60.00%)	49 (72.06%)	0.149
Positive	24 (40.00%)	19 (27.94%)	
eCura scores			
Low risk	47 (78.33%)	31 (45.59%)	0.001
Intermediate risk	12 (20.00%)	34 (50.00%)	
High risk	1 (1.67%)	3 (4.41%)	

M, confined to the mucosa; SM1, depth of invasion from the muscularis mucosae <500 μm; SM2, depth of invasion from the muscularis mucosae ≥500 μm.

### 3.2 Independent risk factors for LNM/local cancer residue and cancer recurrence/cancer-specific mortality

The additional surgery patients were divided into two groups according to the presence or absence of LNM (n = 3)/local cancer residue (n = 8) (**Table 2**), and the follow-up patients were divided into two groups according to the presence or absence of cancer recurrence (n = 4)/cancer-specific mortality (n = 1) (**Table 3**). The differences in basic characteristics of the patients and pathological features after ESD were compared between the two groups.

**Table 2:** Univariable analysis and multivariable logistic regression analysis for LNM/local cancer residue.

	Univariable			Multivariable		
	OR	95% CI	p	OR	95% CI	p
Age < 65	0.15	0.02–1.24	0.08			
Tumor location (upper third)	7.57	1.54–37.29	0.01	54.99	2.52–120.21	0.01
Tumor size (a > 3 cm)	0.96	0.23–4.09	0.96			
Invasion depth (SM2)	6.19	1.23–31.26	0.03	28.24	2.13–37.46	0.01
Undifferentiated type	2.25	0.44–11.46	0.33			
Lymphovascular invasion positive	0.57	0.11–2.93	0.50			
Vertical margin positive	1.96	0.53–7.31	0.32			

LNM, lymph node metastasis.

**Table 3:** Univariable analysis and multivariable logistic regression analysis for cancer recurrence/cancer-specific mortality.

	Univariable		
	OR	95% CI	p
Age < 65	1.74	0.29–10.52	0.55
Tumor location (upper third)	0.41	0.04–3.89	0.44
Tumor size (a > 3 cm)	1.89	0.30–12.01	0.50
Invasion depth (SM2)	0.93	0.15–5.70	0.94
Undifferentiated type	0.15	0.02–1.18	0.07
Lymphovascular invasion positive	1.52	0.15–15.79	0.73
Vertical margin positive	3.78	0.65–21.98	0.14

We found that there were significant differences in upper third location (OR = 54.99, 95% CI: 2.52–120.21, p = 0.01) and SM2 invasion (OR = 28.24, 95% CI: 2.13–37.46, p = 0.01) between patients with and without LNM/local cancer residue among the additional surgery group, while there was no statistically significant difference between patients with and without cancer recurrence/cancer-specific mortality among the follow-up group.

### 3.3 LNM/local cancer residue in the surgical specimens

**Table 4** shows that there were three (4.41%) patients (one in the low-risk group, one in the intermediate-risk group, and one in the high-risk group) with LNM and eight (11.76%) patients (three in the low-risk group, four in the intermediate-risk group, and one in the high-risk group) with local cancer residue in the additional surgery group. Note that the eCura scoring system was a significant factor for LNM (p = 0.044), while local cancer residue in each risk category was not significantly different (p = 0.478) in the three groups.

**Table 4:** Clinical outcomes of EGC patients with non-curative ESD who underwent radical surgery.

		Radical surgery (total number = 68)			
		Low risk (n = 31)	Intermediate risk (n = 34)	High risk (n = 3)	p
LNM	Positive	1 (3.23%)	1 (2.94%)	1 (33.3%)	0.044
	Negative	30 (96.77%)	33 (97.06%)	2 (66.6%)	
Local cancer residue	Positive	3 (9.68%)	4 (11.76%)	1 (33.3%)	0.478
	Negative	28 (90.32%)	30 (88.24%)	2 (66.6%)	

EGC, early gastric cancer; ESD, endoscopic submucosal dissection; LNM, lymph node metastasis.

### 3.4 Cancer recurrence and cancer-specific mortality in the follow-up group

**Table 5** shows that four patients experienced cancer recurrence (one in the low-risk group and three in the intermediate-risk group) in the follow-up group. The eCura scoring system was a significant factor for cancer recurrence (p = 0.017). One patient died of gastric cancer recurrence after 26 months of follow-up. In our study, all cases of cancer recurrence were local recurrence.

**Table 5:** Cancer recurrence and cancer-specific mortality in follow-up group categorized according to the three risk categories of the eCura system.

		Follow-up group (n = 60)			p
		Low risk	Intermediate risk	High risk	
Total number		47	12	1	
Cancer recurrence	Positive	1 (2.13%)	3 (25%)	0 (0%)	0.017
	Negative	46 (97.87%)	9 (75%)	1 (100%)	
Cancer-specific mortality	Positive	0 (0%)	1 (8.33%)	0 (0%)	0.131
	Negative	47 (100%)	11 (91.67%)	1 (100%)	

**Figure 2:**
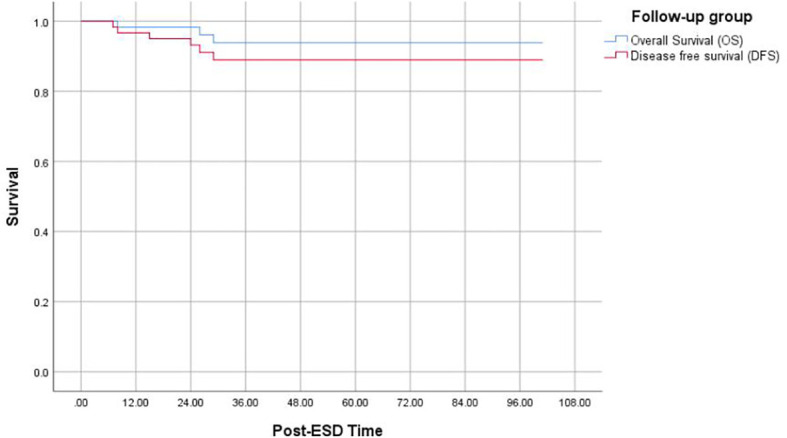
Kaplan–Meier curves for determination of the 5-year OS of EGC after non-curative ESD undergoing simple follow-up. OS, overall survival; EGC, early gastric cancer; ESD, endoscopic submucosal dissection.

**Figure 3:**
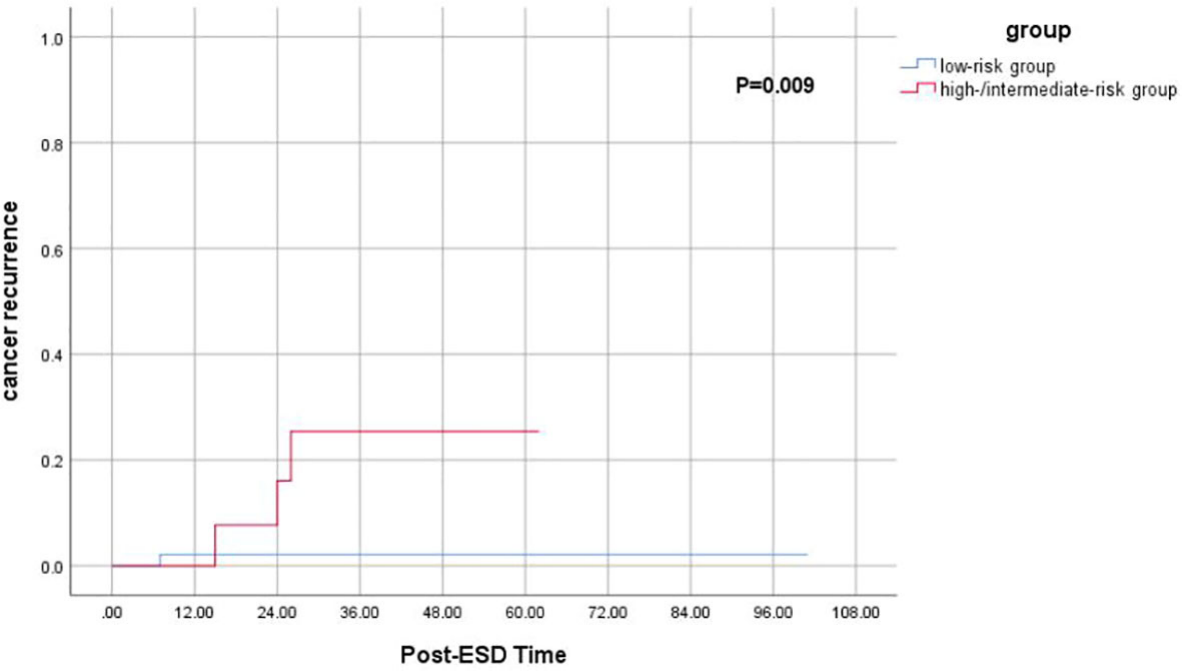
Kaplan–Meier curves for determination of the rate of cancer recurrence for EGC patients after non-curative ESD undergoing simple follow-up. EGC, early gastric cancer; ESD, endoscopic submucosal dissection.

Among 60 follow-up patients, for a median duration of 36 months (range 15–101 months), one patient died of gastric cancer, and two patients died of other diseases. Therefore, the 5-year OS and DFS rates were 93.8% and 88.9%, respectively (**Figure 2**). After applying the “eCura system”, it is obvious that the rate of cancer recurrence in the high/intermediate-risk group was significantly higher than in the low-risk group (p = 0.009) (**Figure 3**).

### 3.5 Details of patients with LNM

**Table 6** shows details of the three male patients with LNM. According to the risk category, one patient was assigned to the low-risk group, one was assigned to the intermediate-risk group, and one was assigned to the high-risk group. All patients contained UD-type histology components, and two patients had a mixed with moderately differentiated adenocarcinoma. In most patients, we found that for a tumor size >3 cm, the depth of tumor invasion was SM2, and the horizontal margins (HMs) were positive.

**Table 6:** Clinicopathological characteristics of the three patients with LNM.

Patient		1	2	3
Endoscopic submucosal dissection	Sex/age (years)	M/56	M/64	M/53
	Location	Middle	Low	Low
	Size (a)	2.3 cm * 1.5 cm * 1.2 cm	4.0 cm * 4.0 cm	3.1 cm * 2 cm * 1.5 cm
	Macroscopic type	O-IIc	O-IIa	0-I
	Ulcer	Absent	Absent	Absent
	Depth of tumor invasion	SM2	SM2	M
	Histology	Mixed-type tubular adenocarcinoma (tub2 > por > pap)	Signet-ring cell carcinoma	Mixed-type tubular adenocarcinoma (tub2 > por > pap)
	Lymphovascular invasion	Negative	Negative	Positive
	Histological margins	Negative	HM and VM positive	HM positive
	eCura scores	1 (low risk)	3 (intermediate risk)	5 (high risk)
Gastrectomy	Local cancer residue	Negative	Negative	Positive
	LNM	1/11	2/11	1/9

HM, horizontal margin; VM, vertical margin; LNM, lymph node metastasis.

## 4 Discussion

Non-curative ESD is closely related to the risk of local cancer recurrence, LNM, and a poor prognosis. The guidelines recommend open or laparoscopic surgical resection due to the clear risk of LNM. However, Oda et al. (9) divided non-curative patients into those undergoing additional gastrectomy (n = 144) and those only undergoing a simple follow-up (n = 82); LNM was identified in 6.3% of patients, with a 5-year disease-specific survival (DSS) rate of 97.8% and a median follow-up period of 4 years. Eriko et al. reported that cancer-specific survival (CSS) did not differ significantly between patients in the additional surgery group and the observation group (10). In addition, as the average life expectancy and the elderly population or the number of patients with severe coexisting diseases increase, further treatment decisions should be made after assessing the risks of recurrence of stomach cancer and LNM.

Therefore, the risk stratification of LNM in patients undergoing non-curative ESD for EGC is needed for appropriate individualized therapeutic strategies. Hatta et al. recently established the “eCura” scoring system for predicting the CSS of patients with non-curative ESD. Cancer-specific mortality and cancer recurrence were regarded as surrogates for LNM by them. They included 1,969 patients who underwent non-curative ESD for EGC at 19 institutions, and patients were divided into radical surgery (n = 1,064) and follow-up (no additional treatment, n = 905) groups. The authors found that the eCura system is a useful aid for selecting the appropriate treatment strategy after non-curative ESD for EGC after they applied it to these patients, but applying this system to patients with UD-type EGC needed to be carried out with caution (11). In addition, Hirotaka et al. found that (12) the eCura scoring system was the only significant factor for cancer residue status.

We conducted a retrospective analysis of 128 patients who underwent non-curative ESD in China, analyzed the independent risk factors for LNM/local cancer residue, and verified the application value of the “eCura” system for the risk stratification of LNM in patients undergoing non-curative ESD for EGC and evaluated whether this system can contribute to the selection of patients requiring radical surgery. In addition, we explored whether other risk factors affect the prognosis of patients with non-curative ESD. Our study revealed three (4.41%) patients with LNM and four (6.67%) patients with cancer recurrence. According to our retrospective study, for most patients who undergo non-curative ESD for EGC, a close follow-up may be a course of action for those who have an advanced age, those unable and/or unwilling to undergo surgery, and so on. We found that tumor location in the upper third and SM2 invasion led to a higher risk of LNM/local cancer residue in patients with non-curative ESD. As previously reported, EGC at a proximal location might have a higher rate of LV and deep SM invasion than at the antrum or angle. Researchers explained that it was difficult to make early detection for tumors located at the mid or upper third of the stomach because EGC can be hidden between gastric folds, especially when they are located at the greater curvature (13). By applying the scoring system, we found that the high-risk group (33.3%) had a higher risk of LNM than the low-risk (3.23%) and intermediate-risk (2.94%) groups (p = 0.044). The eCura scoring system was the significant factor for cancer recurrence (p = 0.017). In addition, the rate of cancer recurrence in the high/intermediate-risk group was significantly higher than in the low-risk group (p = 0.009). Therefore, salvage surgery can be suggested in intermediate-risk and high-risk patients.

The well-known risk factors for LNM include the presence of LVI, a large tumor size (>3 cm), VM positivity, SM invasion, and the UD histologic type. In our study, among three patients with LNM, two cases were mixed-type tumors with sizes larger than 2 cm. There were significantly lower curative resection rates in mixed predominantly differentiated type (41.7 *vs.* 92.0%; p < 0.0001)/mixed predominantly UD type (35.7 *vs.* 75.9%; p = 0.0002) than pure histologic counterparts (14). A higher LNM incidence rate and more aggressive behavior had been reported in mixed-type tumors than in other histological types (15). A logistic regression analysis revealed that histologic types of mixed (OR, 2.360) and pure UD (OR, 1.657) were the independent risk factors for LNM (16). Multivariate analysis (17) revealed that tumor histology was also significantly associated with LNM in mucosal cancers, the rates of which were higher in mixed-type tumors (6.3%) than in the other two types (p = 0.005). Mixed-type histology EGC frequently indicates larger size, deeper invasion, and higher rates of LVI/LNM than pure-type EGC. Among three LNM patients, one case was a mixed-type tumor with SM2 invasion (score of 1 point) and another was a case of positive LVI (score of 4 points). LVI was the most important risk factor for LNM (18) and also was an indispensable predictor of cancer recurrence and cancer-specific death in the follow-up group. Our data show that the incidence of LNM in the LVI-positive group was 20%, and many other studies have reached similar conclusions. Kim (18) found that the incidence rate of LNM in the LVI-positive group was 21% (9/35), and Sekiguchi et al. (19) reported that the incidence of LNM in LVI positivity with mucosal invasion was 29.2% (7/24). Thus, even if the tumor is confined to the mucosa, LNM is a risk when LVI is positive. However, we need to recognize the limitation that it was difficult to detect LVI in UD tumors even with immunohistochemical staining. One case was signet-ring cell carcinoma (SRC), while the tumor size was 4 cm * 4 cm. SRC is a poorly differentiated cancer that is believed to show a poor prognosis and aggressive behavior. Sun et al. found that in 91 patients with SRCs, only 5 had LNM, and all the lesions in the patients with LNM were greater than 3 cm. Therefore, SRC histology itself was not a risk factor for LNM, but SRCs with large sizes showed high LNM. In addition, SRCs with mixed histology showed more SM invasion, a large size, and a high rate of LNM (20). Therefore, endoscopic surgery should be limited to the differentiated type of invasive SM without histological heterogeneity (21, 22). The case in our study exceeded the indications for ESD due to the precise endoscopic prediction of EGC (i.e., tumor depth/size is sometimes difficult to assess before treatment). Ryu et al. (23) reported that it was difficult to predict the range of UD cancers using endoscopy, and the study also revealed that the proliferative zone of UD intramucosal cancer was always located in the intermediate zone of the mucosa among the 12.5% of depressed-type EGC and 85.7% of flat-type EGC; hence, the surface was always covered with normal mucous membrane cells. As a result, UD intramucosal carcinoma tends to spread more widely than its general appearance. Through logical regression analysis, Kang et al. (24) revealed that scar deformity, nodular surface appearance, and surface depression tended to invade SM2 for early SRC (OR 3.4, 5.9, 6.0; p < 0.05). For UD EGC patients with non-curative ESD, Jie-Hyun Kim et al. found that recurrence-free survival (RFS) was longer in the additional surgery after ESD/surgery group than in the ESD group (25).

Positive VM was also a risk factor for LNM, with a score of 1 point. A meta-analysis (26) involving 1,720 patients with EGC indicated that positive VM (p < 0.001) was significantly associated with LNM, while HM and ulceration were not identified as risk factors associated with LNM. Japanese Gastric Cancer Treatment Guidelines (27) (2018) suggested that when it is of a histologically differentiated type and fulfills other criteria to be classified into curative ESD but is either not resected en bloc or has positive HM, repeat ESD, surgical resection, close observation expecting a burn effect of the initial ESD, and endoscopic coagulation using a laser or argon-plasma coagulator could be good selections since the risk for harboring LNM was relatively low. Of the three LNM patients in this study, two had tumor sizes larger than 3 cm (score of 1 point). According to research (28), the tumor size incidence rate of LNM was as follows: under 2 cm, 14% (64 patients); 2.1–3.0 cm, 27% (68 patients); and over 3 cm, 31% (96 patients).

Cancer-specific mortality and cancer recurrence were also important indicators for evaluating the prognosis of patients with EGC. In many studies, LVI positivity, a large tumor size, VM positivity, SM invasion, and so on were risk factors. Unfortunately, we found no independent risk factors for cancer recurrence/cancer-specific mortality. In our study, four male patients presented local cancer recurrence after follow-ups of 26, 7, 15, and 24 months. The rate of cancer recurrence was higher in SM2 (8.70%, 2/23) and SM1 (7.69%, 1/13) than in M (4.35%, 1/23). The incidence rate of cancer recurrence in the mixed-type tumor, UD-type tumor, VM positivity, and HM positivity was 8.7% (2/23), 33.3% (2/6), 27.27% (3/11), and 4.17% (1/24), respectively. In addition to VM positivity, HM positivity is an important risk factor for local recurrence. A total of 11,796 ESD cases were enrolled, and 229 patients (2%) had positive horizontal or indeterminable margins (29); during 6 months of follow-up period, 27 (21%) cases experienced cancer recurrence.

Although patient 2 with LNM did not receive a score in the eCura system, it was a UD type with a size larger than 2 cm. Waku Hatta et al. did not include the histopathology in the eCura system leading to selection bias, so the risk of LNM in UD-type tumors cannot be predicted precisely by the eCura system. We found that the LNM and tumor recurrence occurred with the UD/mixed-type tumor in the low-risk group. The phenomenon indicated that caution in the use of this scoring system is needed for patients with UD-type EGC, and the depth of invasion, tumor size, UD/mixed-type, LVI-positivity, and so on should be considered.

Our study has several limitations. First, the sample size and follow-up periods were limited, the validation ability of the factor UD type was slightly weak, and bias may exist in the analysis of risk factors for LNM and cancer recurrence. However, the data showed that the risk of LNM in the high-risk group tended to be the highest, and in the low-risk group, it tended to be the lowest. Second, it was a retrospective study based on medical records, and its retrospective design introduces the potential for selection bias. To minimize this bias, we included almost all patients with EGC treated with ESD identified in the database. Third, because this study was conducted at seven institutions, endoscopic diagnosis before ESD, ESD procedures, and histopathological assessment were performed by independent endoscopists and pathologists at each institution.

In conclusion, we revealed that the eCura system is likely helpful for the risk stratification of LNM for Chinese patients. For non-curative ESD, salvage surgery and LN dissection can be suggested in intermediate-risk and high-risk patients; a close follow-up might be a management option in low-risk patients who have an advanced age and in those with severe concomitant disease and/or unwilling or unable to accept additional gastrectomy. However, if there are mixed-type or UD-type tumors, we suggest undergoing salvage surgery because a high risk of LNM may exist, especially when the depth and transverse invasion of the tumor are difficult to determine. It is necessary to improve the accuracy of preoperative pathological indications and strictly grasp the indications of ESD so as to approve the curative ESD and obtain a better prognosis.

## Data availability statement

The raw data supporting the conclusions of this article will be made available by the authors, without undue reservation.

## Ethics statement

The study was reviewed and approved by the Ethics Committee of The First Affiliated Hospital of Zhejiang Chinese Medical University (IRB no. 2019-KL-110-01). Informed consent was obtained from all subjects. All methods were carried out in accordance with relevant guidelines and regulations.

## Author contributions

All authors helped to perform the research. C-QJ and JZ contributed to manuscript writing, data acquisition, and analysis. X-YD, L-LY, G-LY, X-JZ, J-WS, YY, and BJ contributed to data acquisition and analysis. C-LZ and BL contributed to supervision or mentorship. BL also contributed to drafting, conception, and design. BL takes responsibility for the authenticity and accuracy of the present study. All authors contributed to the article and approved the submitted version.
